# Impairment of Smooth Pursuit as a Marker of Early Multiple Sclerosis

**DOI:** 10.3389/fneur.2016.00206

**Published:** 2016-11-21

**Authors:** Nathaniel Lizak, Meaghan Clough, Lynette Millist, Tomas Kalincik, Owen B. White, Joanne Fielding

**Affiliations:** ^1^Department of Medicine, University of Melbourne, Melbourne, VIC, Australia; ^2^Monash School of Medicine, Monash University, Melbourne, VIC, Australia; ^3^School of Psychological Sciences, Monash Institute of Cognitive and Clinical Neurosciences, Monash University, Melbourne, VIC, Australia; ^4^Department of Neurology, Royal Melbourne Hospital, Melbourne, VIC, Australia

**Keywords:** multiple sclerosis, smooth pursuit, clinically isolated syndrome, ocular motor system, neuro-ophthalmology

## Abstract

**Background:**

Multiple sclerosis (MS) is a diffuse disease that disrupts wide-ranging cerebral networks. The control of saccades and smooth pursuit are similarly dependent upon widespread networks, with the assessment of pursuit offering an opportunity to examine feedback regulation. We sought to characterize pursuit deficits in MS and to examine their relationship with disease duration.

**Methods:**

Twenty healthy controls, 20 patients with a clinically isolated syndrome (CIS), and 40 patients with clinically definite MS (CDMS) participated. Thirty-six trials of Rashbass’ step–ramp paradigm of smooth pursuit, evenly split by velocity (8.65°, 17.1°, and 25.9°/s) and ramp direction (left/right), were performed. Four parameters were measured: latency of pursuit onset, closed-loop pursuit gain, number of saccades, and summed saccade amplitudes during pursuit. For CDMS patients, these were correlated with disease duration and Expanded Disability Status Scale (EDSS) score.

**Results:**

Closed-loop pursuit gain was significantly lower in CIS than controls at all speeds. CDMS gain was lower than controls at medium pursuit velocity. CDMS patients also displayed longer pursuit latency than controls at all velocities. All patients accumulated increased summed saccade amplitudes at slow and medium pursuit speeds, and infrequent high-amplitude saccades at the fast speed. No pursuit variable significantly correlated with EDSS or disease duration in CDMS patients.

**Conclusion:**

Smooth pursuit is significantly compromised in MS from onset. Low pursuit gain and increased saccadic amplitudes may be robust markers of disseminated pathology in CIS and in more advanced MS. Pursuit may be useful in measuring early disease.

## Introduction

Multiple sclerosis (MS) is characterized by chronic episodic and progressive inflammatory degeneration of the central nervous system, leading to significant degradation of quality of life (1). While episodes of neurological damage to “eloquent” regions producing discrete radiological lesions are considered the hallmark of MS (2), extensive damage to neurological networks can be observed from onset (3–9). However, quantitative MRI techniques used to establish this pathology are expensive and time consuming (10), and do not directly mirror functional impairment. Similarly, standard clinical assessment tools remain fairly insensitive to subtle network injury, generating a need for novel ways of measuring such pathology.

Previous work done by our group has highlighted the utility of ocular motor assessment in quantifying pathological changes from the earliest inception of the disease, known as a clinically isolated syndrome (CIS), and in more advanced MS (11–17). This work has focused on the assessment of saccades, rapid eye movements that center a visual target onto the fovea, and implicate a widespread neuronal network (11, 18). However, other outputs of the ocular motor system have not been investigated as extensively.

Smooth pursuit is another form of eye movement that enables tracking of objects moving in space. The smooth pursuit system incorporates closed-loop neuronal systems, continuously utilizing real-time negative feedback, for the critical task of maintaining optimal fixation of a target in motion by aligning it with the fovea (19). While pursuit has traditionally been viewed as a reflexive behavior under feedback control, recent evidence suggests it is reliant on neural networks similar to those involved in saccade generation, with pursuit and saccades merely different outcomes of the same sensorimotor system (20). However, there are fundamental differences in their execution: while saccades are a single pseudo-ballistic movement, pursuit requires constant regulation by feedback loops (19). Assessing smooth pursuit allows not only the investigation of movement generation, as with saccades, but also affords a novel opportunity to examine the integrity of combined visual and motor feedback loops, and the impairment of feedback control.

Consequently, pursuit may offer an independent measure of widespread network damage in MS and possibly provide a differential marker in staging disease. Studies have investigated pursuit in MS, with evidence demonstrating poorer pursuit gain (21, 22). However, there is currently no understanding about when pursuit deficits first manifest, and whether these deficits deteriorate with increasing disease activity, providing a marker for monitoring disease.

We aimed to characterize pursuit failure in MS, establish when such deficits originate, and determine their evolution over the course of disease.

## Patients and Methods

### Participants and Recruitment

Twenty patients with CIS, 40 patients with clinically definite MS (CDMS), and 20 neurologically healthy controls participated. CIS patients were included based on an initial neurological episode, suggestive of MS, with MRI evidence of demyelination. CDMS patients were included based on a definitive diagnosis of MS as per the McDonald criteria (2). Healthy controls were recruited from the community; exclusion criteria for this group included a history of traumatic head injury, neurological or psychiatric disorder, drug abuse, or regular intake of psychoactive drugs.

All patients were recruited through either Cabrini or Royal Melbourne Hospitals, Australia, and tested at the latter. Ethics approval was granted by the Melbourne Health Human Research Ethics Committee, and all participants gave their written informed consent prior to inclusion, in accordance with the declaration of Helsinki.

### Smooth Pursuit Testing

All participants were tested using Rashbass’ step–ramp paradigm of smooth pursuit at constant velocity (23). At the time of testing, no CIS or CDMS patients were suffering from exacerbated symptomatology or demonstrated any evidence of active disease, and all participants had adequate visual acuity to perform the task. Testing was conducted as part of a larger set of ocular motor assessments, the results of which have been published elsewhere (12, 13).

Participants were seated in a darkened room, directly in front of the display monitor, at a distance of 840 mm. Horizontal ocular displacement was recorded using the EyeLink II dark pupil, video-oculography system (SR-Research Ltd., Mississauga, ON, Canada). This is a high-resolution (noise limited at <0.01°), high-acquisition rate (500 Hz) system, with three miniature cameras mounted on a headband. All screen-based stimuli were generated using Experiment Builder (version 1.10.165), and displayed on a 22″ CRT monitor.

**Figure 1:**
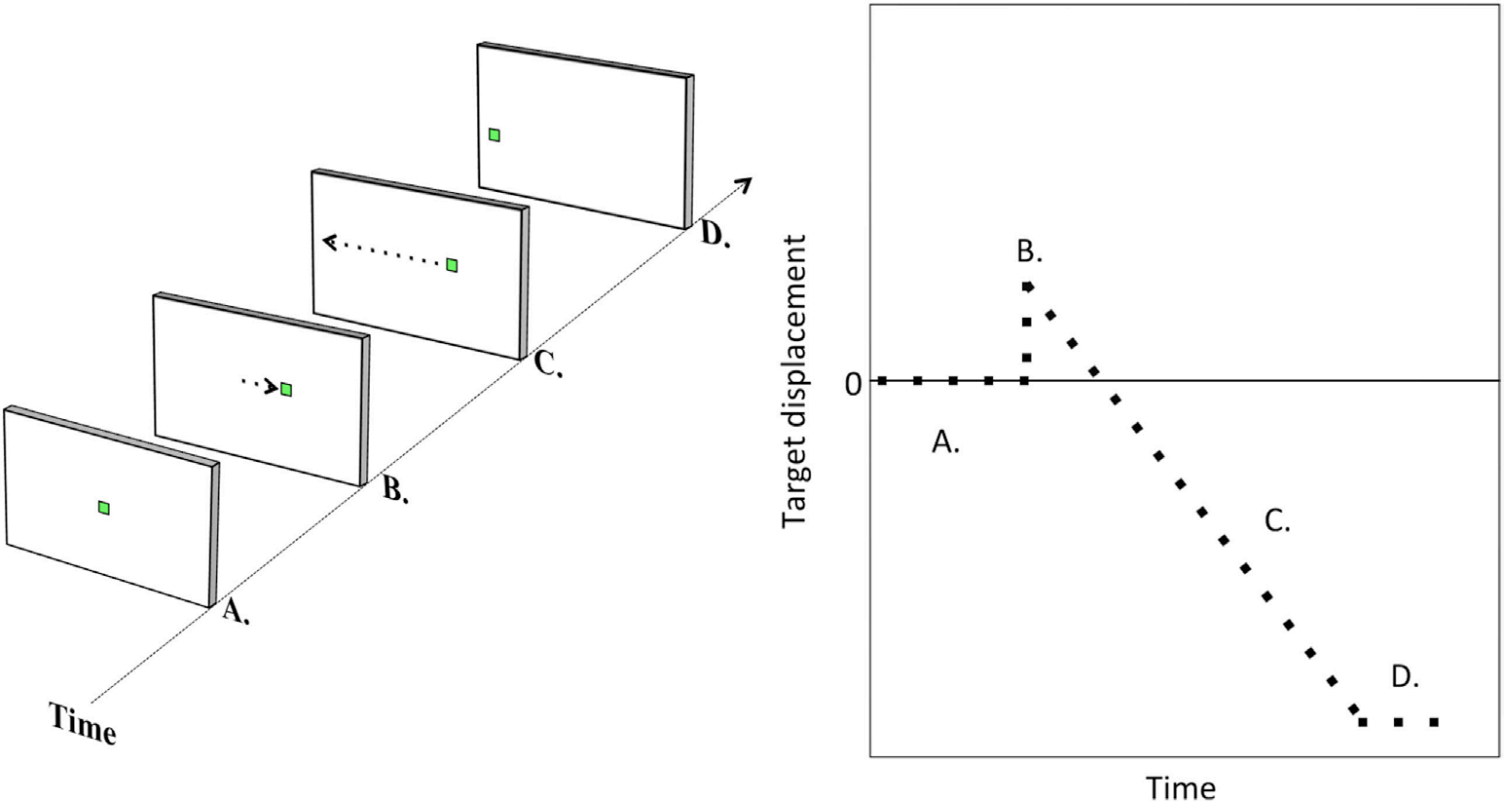
**Task illustration**. Illustration of a step–ramp pursuit trial. The target is initially central (A). It is then suddenly replaced by a target either left or right of center [target step; (B)]. The target then moves at constant velocity to the other side of the screen [target ramp; (C)] until reaching a position of 10° either left or right of center (D).

Each trial of the step–ramp paradigm commenced with the presentation of a fixation target centrally: a 20 × 20 pixel white square ring, subtending 0.5° visual angle. The fixation target was maintained for a variable period: 1000, 1500, 2000, or 2500 ms, equally weighted between ramp speeds and directions. This fixation target was then replaced by the task target, a small green square 21 × 21 pixels and subtending approximately 0.5° visual angle. The green square appeared either right or left of the fixation target [i.e., the “step” (23)] and then moved in the opposite direction at a constant velocity [the “ramp” (23)] through the center position and toward the opposite side of the screen. The trial terminated when the target reached the 10° position either left or right of center. Participants were instructed to follow the target as it moved through the screen. Figure 1 illustrates the task.

Each participant performed 36 individual trials of this paradigm, broken up into 3 blocks of 12, with a brief rest break between each block. Trials were evenly split by ramp direction (left/right) and velocity: 8.65°, 17.1°, and 25.9°/s. Greater step magnitudes were utilized at greater ramp velocities (1.4°, 2.69°, and 4.12°, respectively), calculated to provide a latency of about 160 ms (24) for the target to reach the center of the screen from the initial eccentric position. This was intended to lower the likelihood of a saccade being triggered at pursuit initiation. Trials were arranged in a randomized order with regard to ramp speed and direction, which was identical for all participants.

### Data Analysis

Latency of pursuit onset, closed-loop pursuit gain, and the number and amplitudes of saccades made during each trial were examined. Analysis of each of these variables was conducted separately at each pursuit speed (8.65°, 17.1°, and 25.9°/s).

Pursuit onset was manually determined as the point at which the eye commenced net movement in the direction of the target, with no or only minor movement in the opposite direction. Latency was calculated as the time from target step to pursuit onset. Excluded from the calculation of latency were all trials beginning with a saccade, or where pursuit onset was delayed by saccades, blinks, and purposeless eye movements (e.g., square wave jerks), or where onset could not be identified due to ocular drift.

Closed-loop pursuit gain was calculated as the ratio of target to eye velocity during closed-loop pursuit (i.e., smooth tracking of the target with online correction). This was evaluated during a single continuous period of smooth pursuit within each trial, excluding any saccades, blinks, and square wave jerks, where the participant displayed their most optimal pursuit (i.e., greatest gain). To ensure that this was not an open-loop (i.e., predictive) smooth eye movement, this period was always selected at least 200 ms after pursuit onset. Rarely, if less than 20 ms of pursuit was available, usually due to excessive blinking, trials were removed from the analysis. Through this calculation, a gain value of 1 was effectively “perfect” pursuit, and lower the gain value, the worse the participant’s ability to pursue the target.

Finally, saccade counts and amplitudes were obtained during each step–ramp trial. All saccades performed from target step through to the end of the trial (when the target reached the 10° position) were included. Automated saccade detection identified periods of the segment where velocity magnitude (i.e., independent of positive or negative sign) exceeded background velocity by more than 5°/s and then declined back to below this level. If the peak velocity between these two points was <45°/s (likely baseline drift) or >400°/s (likely blink or artifact), the section was automatically discarded. All traces were visually inspected to identify additional saccades not detected automatically, and to remove any blinking, square wave jerks, or artifact incorrectly labeled as a saccade (19). Saccade counts simply reflected the number of saccades made during the trial. Summed saccade amplitudes were calculated as the sum of amplitudes of all saccades performed during each pursuit trial, as a measure of saccadic interruption of pursuit.

Saccades measured were the combination of all catch-up, back-up, and task-inappropriate saccades performed by participants.

### Statistical Analysis

Statistical analysis was carried out using R version 3.1.0 (http://www.R-project.org). All hypotheses were tested at the two-tailed 0.05 level of statistical significance. Planned comparisons between individual groups were conducted using Welch’s *t*-tests (or, where distributions were non-normal, Wilcoxon rank-sum tests) to determine differences in pursuit latency, gain, and saccade counts and summed amplitudes. These variables were also correlated with disease duration and Expanded Disability Status Scale (EDSS) step in the CDMS group, using Pearson’s (*r*) correlations or Spearman’s rho, depending on distributions.

## Results

Demographic and clinical characteristics of the participants are summarized in Table 1. CDMS patients were significantly older than the other two groups, which would be expected given that, by definition, they have suffered MS for longer than CIS patients. However, no relationships were found, for any group, between markers of pursuit performance and participant age. Consequently, age was not considered as a factor.

**Table 1:** **Participant characteristics**.

	CDMS	CIS	Controls
Number	40	20	20
Age, years (mean ± SD)	43.0 ± 11.4	33.3 ± 8.2	35.1 ± 13.1
Gender (number female, % female)	37, 93	16, 80	13, 65
EDSS (mean ± SD)	1.3 ± 1.4	–	–
Disease duration, years (mean ± SD)	8.0 ± 5.9	–	–

*EDSS, Expanded Disability Status Scale; CDMS, clinically definite multiple sclerosis; CIS, clinically isolated syndrome*.

**Figure 2:**
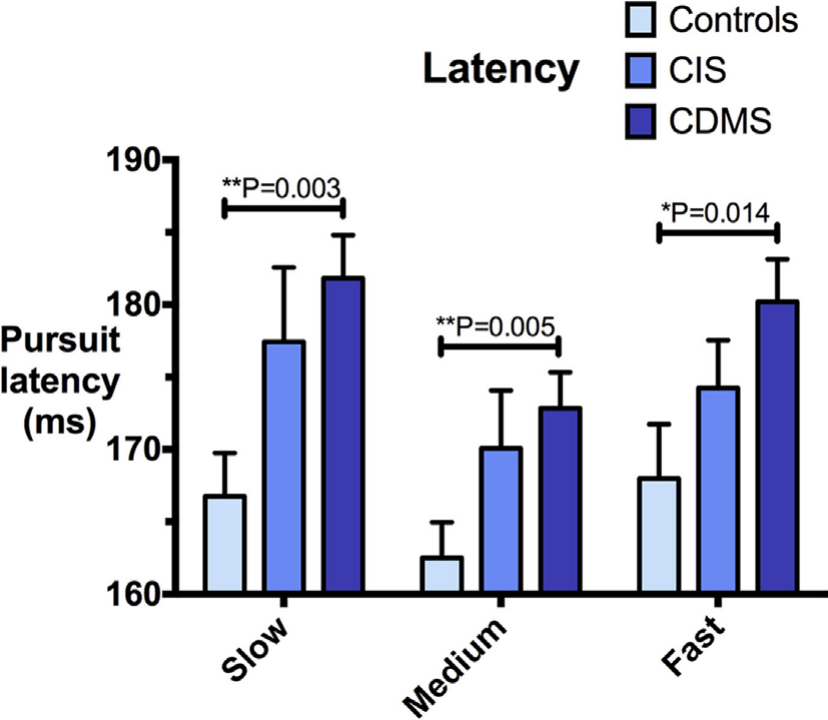
**Comparison of pursuit latency**. Differences in pursuit latency (milliseconds) at slow (8.65°/s), medium (17.1°/s), and fast (25.9°/s) pursuit speeds. Bars: mean latency. Error bars: SEM. *P < 0.05, **P < 0.01, ***P < 0.001.

**Figure 3:**
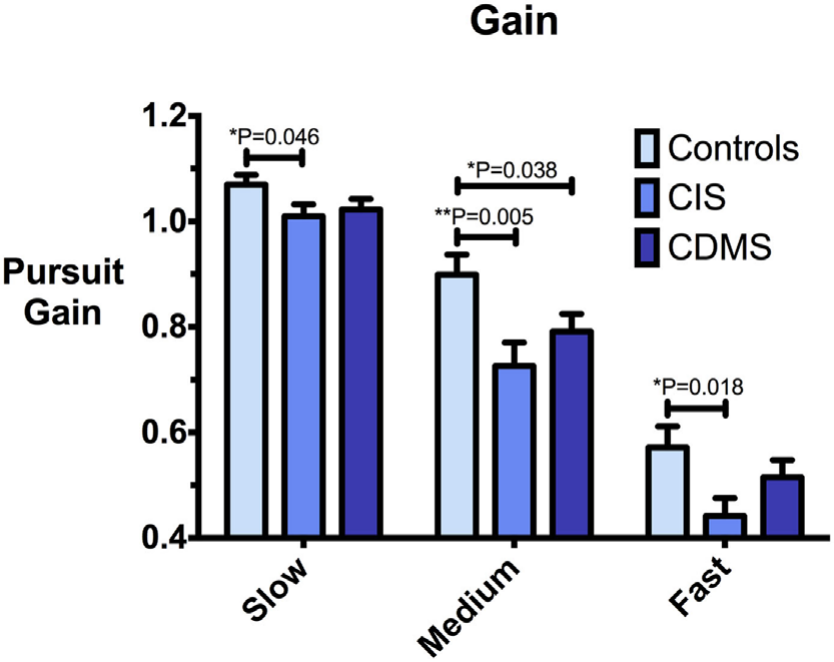
**Comparison of pursuit gain**. Differences in pursuit gain at slow (8.65°/s), medium (17.1°/s), and fast (25.9°/s) pursuit speeds. Bars: mean gain. Error bars: SEM. *P < 0.05, **P < 0.01, ***P < 0.001.

**Figure 4:**
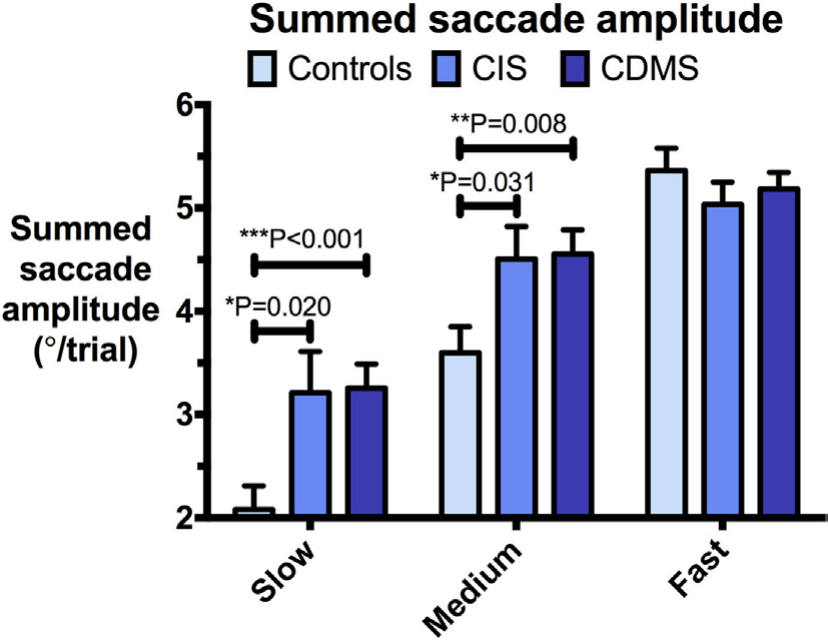
**Comparison of summed saccade amplitudes**. Differences in summed saccade amplitudes (°/trial) at slow (8.65°/s), medium (17.1°/s), and fast (25.9°/s) pursuit speeds. Bars: mean summed saccade amplitude. Error bars: SEM. *P < 0.05, **P < 0.01, ***P < 0.001.

**Figure 5:**
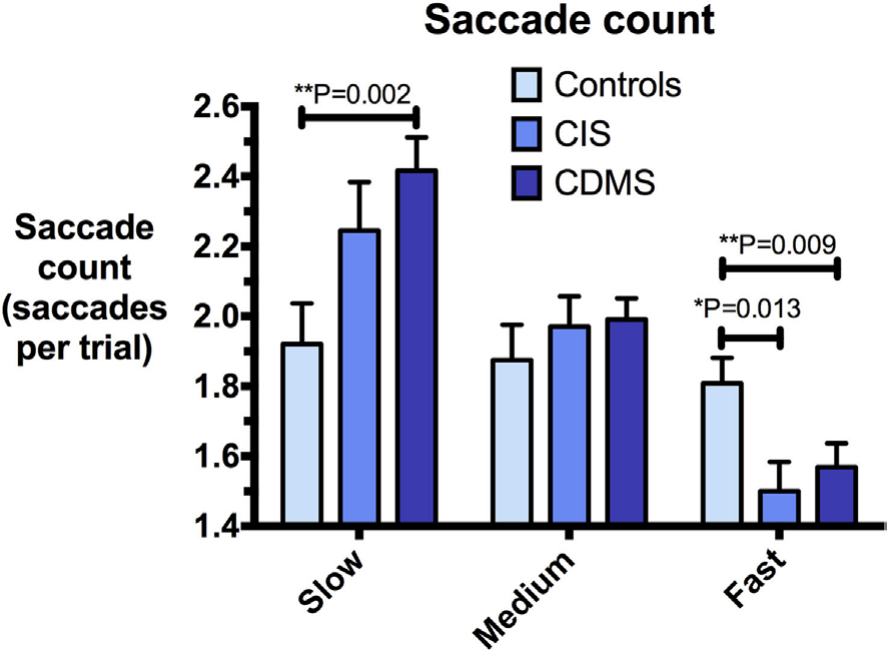
**Comparison of saccade counts**. Differences in saccade counts (saccades/trial) at slow (8.65°/s), medium (17.1°/s), and fast (25.9°/s) pursuit speeds. Bars: mean saccade count. Error bars: SEM. *P < 0.05, **P < 0.01, ***P < 0.001.

Group means and differences for all variables are demonstrated in Figures 2–5, and statistical results for all comparisons between controls and patient groups are shown in Table 2.

**Table 2:** **Pursuit comparisons**.

Variable	Comparison with controls	
	CIS *t*(DF)/*W*	CDMS *t*(DF)/*W*
**Pursuit gain at**		
−8.65°/s	*t*(36.5) = 2.07*	*W* = 476
−17.1°/s	*t*(37.2) = 2.97**	*t*(46.3) = 2.13*
−25.9°/s	*t*(37.0) = 2.48*	*W* = 482
**Pursuit latency (ms) at**		
−8.65°/s	*t*(30.6) = 1.79	*W* = 587**
−17.1°/s	*t*(31.4) = 1.61	*t*(51.6) = 2.97**
−25.9°/s	*t*(37.3) = 1.25	*t*(41.7) = 2.56*
**Summed saccade amplitudes (°/trial) at**		
−8.65°/s	*t*(30.3) = 2.45*	*t*(51.5) = 3.61***
−17.1°/s	*t*(36.3) = 2.25*	*t*(48.3) = 2.77**
−25.9°/s	*t*(38.0) = 1.07	*t*(39.7) = 0.66
**Saccade count (saccades per trial) at**		
−8.65°/s	*t*(37.0) = 1.80	*t*(43.3) = 3.30**
−17.1°/s	*W* = 184	*W* = 354
−25.9°/s	*W* = 292*	*W* = 567**

*Statistical values for comparisons between patient groups (CIS and CDMS) and controls. Where distributions were normal, *t*-tests were utilized, and the value of *t* and the degrees of freedom (DF) are displayed. Where assumptions were violated, Wilcoxon rank-sum tests were used instead, and the value of *W* is displayed*.

*P < 0.05.

**P < 0.01.

***P < 0.001.

*CIS, clinically isolated syndrome; CDMS, clinically definite multiple sclerosis*.

### Pursuit Latency

Clinically definite MS patients demonstrated significantly greater latencies than healthy controls at all pursuit velocities. Although CIS patients had longer latencies than controls, but shorter than CDMS patients, these trends were not statistically significant. Figure 2 illustrates latency values for all groups.

### Pursuit Gain

At all pursuit speeds, CIS patients exhibited significantly lower closed-loop pursuit gain than healthy controls. At the medium pursuit velocity (17.1°/s), where this difference was most pronounced, CDMS patients also displayed significantly lower gain than controls. At slow pursuit velocity, gain slightly above 1 for all groups implies that eye velocity consistently exceeded target velocity. Figure 3 demonstrates these results.

### Saccades during Pursuit

At slow and medium pursuit velocities (8.65°/s and 17.1°/s), both CIS and CDMS groups demonstrated significantly greater summed saccade amplitudes than healthy controls. CDMS patients also performed more saccades than controls at the slowest velocity. At medium velocity, no differences were observed in saccade counts. At the fastest pursuit velocity (25.9°/s), there were no significant differences in summed saccade amplitudes; however, controls displayed a greater saccade count than both CIS and CDMS groups. These differences are shown in Figures 4 and 5.

Although saccades were initially categorized as catch-up, back-up, and task-inappropriate, catch-up saccades predominated. Indeed, of all saccades made by trial participants, 78.8–87.4% in slow, 95.6–97.0% in medium, and 97.5–98.6% in fast pursuit were of the catch-up type. Of the mean summed saccade amplitudes, the contribution made by catch-up saccades was: 79.2–87.6% in slow, 96.2–98.4% in medium, and 98.7–99.7% in fast pursuit. There were no significant differences between groups in numbers and amplitudes of back-up and task-inappropriate saccades, and comparisons of all saccade types or exclusively of catch-up saccades yielded identical results (latter not shown). Hence, saccade types were collapsed, and the analysis, as reported above, was carried out on all saccades performed.

There were no significant differences between CIS and CDMS groups for any of the pursuit variables (latency, gain, and saccades).

### Correlations with Clinical Variables

There was a near-significant positive correlation between CDMS latency at the slowest velocity (8.65°/s) and EDSS score (Spearman’s rho = 0.31, *P* = 0.055). No other CDMS pursuit variables (gain, latency, and saccade amplitudes and counts) at any velocities correlated with either EDSS or disease duration (data not shown).

## Discussion

While saccadic impairments are now a recognized feature of MS (11), previous research on smooth pursuit in MS has been restricted to descriptions of poor gain and a preponderance of catch-up saccades in patients with long-standing CDMS (21, 22). Our study further characterizes these deficits, and is the first to investigate pursuit at the earliest stages of disease. Using a step–ramp paradigm, we found that closed-loop pursuit gain is lowered in patients with CIS, the first clinically detectable presentation of MS, and remains abnormally low as disease progresses to CDMS. Simultaneously, there is a tendency for increased saccade generation during pursuit, with abnormally elevated saccade amplitudes observed in both CIS and CDMS patients. Pursuit latency is delayed in CDMS.

Our results suggest that smooth pursuit is substantially impaired at the inception of clinical disease (CIS), similar to saccades (12, 13). While saccades and smooth pursuit eye movements are subserved by a largely overlapping neural network (20), pursuit involves an additional level of complexity, involving visual feedback control of pursuit performance; this likely explains the seeming vulnerability of pursuit to minor disruptions to its subserving networks, as likely occurs in CIS.

Indeed, poor pursuit gain in all patients is highly suggestive of impairments in feedback loops (failure of both afferent feedback and the subsequent efferent generation), occurring from disease onset. The observed tendency for catch-up saccades likely reflects a compensatory mechanism for inadequate pursuit, and that when the nexus between feedback regulation and smooth pursuit generation breaks, saccades are generated.

Importantly, we analyzed gain during a short, optimal segment of closed-loop smooth eye movement. This was done with the deliberate intent of assessing the maximal capacity of the system to generate pursuit; saccade amplitudes were used as a marker of overall pursuit maintenance and failure to generate pursuit consistently. Given that reduced gain was observed during the optimal segment of pursuit in this pilot, overall gain should also be examined, which is best evaluated through a sinusoidal task.

Partly, analysis of gain during optimal pursuit may explain why, at slow target velocity, control gain was above 1, and the CIS group displayed a significantly reduced pursuit gain with a mean of 1.01 (which is seemingly normal). However, this also suggests that an optimal smooth eye movement, at this target velocity, is somewhat predictive in nature, explaining why optimal gain was above 1 (i.e., faster than target). Possibly, CIS patients had a lesser ability to perform predictive eye movements at this velocity. Nonetheless, the presence of abnormally elevated saccade amplitudes at slow speed, particularly catch-up saccades, does imply that overall pursuit gain is likely still sub-optimal in both CIS and CDMS patients. The use of sinusoidal tasks to evaluate overall gain may be better able to differentiate poor gain in these patients than testing optimal gain.

Curiously, a lower saccade count at fast target velocities was observed in both CIS and CDMS compared to controls, despite essentially identical saccade amplitudes overall. Patients therefore generated fewer saccades with greater amplitudes. The speed of 25.9°/s is intentionally slightly under the upper limit of normal smooth pursuit (25), and the generation of high-amplitude saccades at this speed indicates a pathological lowering of the maximum pursuit gain. Our control data also imply that frequent low-amplitude saccades are a normal compensatory mechanism at this velocity. Given the close interplay between saccades and pursuit during tracking (26), it is possible that injury to the complex system underlying the selection of different ocular movements has led to an impaired ability to correct position error in CIS and CDMS patients.

Delayed pursuit onset in CDMS patients in comparison to control participants signifies that in addition to feedback insufficiency, the initial generation of pursuit is compromised with disease progression. This is consistent with our previous findings for saccadic eye movements, where prolonged latencies become increasingly evident with longer disease durations (12).

Unexpectedly, we revealed no significant differences in performance between CDMS and CIS patients for any measure, although a number of visible trends suggest differential performance with disease progression. This is possibly a consequence of the relatively low disease burden for our CDMS cohort (mean EDSS 1.3), or of low power. Comparing CIS patients with more advanced CDMS patients may allow for better discrimination between these two groups. Furthermore, correlations between pursuit abnormalities and EDSS scores, non-significant in the present study, may be more informative when conducted over a wider spectrum of disease severity. We intend to do this through our ongoing longitudinal studies.

Overall, our results provide further evidence that select ocular motor measures of performance can reflect clinically undetectable widespread neuronal injury at the earliest stages of MS (27). Given that more than 50% of cerebral circuitry is intimately involved in the control of vision and/or eye movements (28), disruption of these processes in the context of widespread structural damage is not only possible but expected.

Pursuit may therefore provide an inexpensive, fast, and objective tool for detecting widespread pathology in CIS and CDMS, with potential utility in identifying subclinical deficit. While both pursuit gain and saccade amplitudes may be robust markers of disease, it must be noted that gain was assessed solely during optimal pursuit, with segments of poorer pursuit ignored. This may explain why gain was seemingly normal for CDMS at slow and fast velocities, despite abnormal saccade profiles. Because summed saccade amplitude is independent from the subjective selection of an “optimal” pursuit segment, and also extremely quick and easy to calculate, it is likely the most appropriate variable for future clinical applications from those tested in this study. However, overall gain during each trial has the potential to be an effective discriminator, and we intend to examine this through sinusoidal tasks, which we are currently collecting data for. Furthermore, the medium pursuit velocity appears to best discriminate the presence of disease, particularly with respect to gain.

Measurement of pursuit latency, which is abnormally delayed in CDMS patients, may also be of clinical utility. Given the relatively low disease burden of our CDMS cohort, it is possible that increased pursuit latency may provide a physiological marker contributing to staging of disease prior to substantial advances in the EDSS, potentially affording an early indicator for escalating therapy. Indeed, the observed trend for latency to increase as a function of disease stage (Figure 2) suggests that pursuit latency may be a marker of the extent of pathology. Further studies are crucially required to validate the utility of pursuit measures in discerning the presence and/or progression of disease in MS, and to examine if these are indeed beneficial in predicting disease progression before disability accumulation occurs. We are currently collecting the data for such longitudinal studies of individual patients.

It is important to note that we measured pursuit performance at the end of a testing battery including multiple ocular motor tasks. Fatigue may thus be a contributing factor to differential performance between MS patients and controls. The test conductor and the data analyst were not blinded to each participant’s group, possibly increasing the likelihood of detection bias. However, gain and, in particular, saccade amplitudes, are highly objective markers and unlikely to have been significantly influenced by possible bias.

Our study is the first to investigate the relationship between disease stage and pursuit profile, and we have shown that smooth pursuit integrity is already substantially affected in CIS and early CDMS patients. Physiologically, this represents evidence of feedback loop dysfunction at the onset of clinical disease, with impairments in motor generation becoming evident at the early stages of CDMS. Smooth pursuit thus has promising utility as a cost-effective means of detecting early disseminated disease. Longitudinal studies are merited to examine whether pursuit deficits may be useful in predicting future conversion from CIS to CDMS, in monitoring disease activity, and in evaluating the need for therapy.

## Ethics Approval

Ethics approval was provided by the Melbourne Health Human Research Ethics Committee. All participants gave their written informed consent prior to inclusion, in accordance with the declaration of Helsinki.

## Author Contributions

NL conducted the analysis, and drafted and revised the manuscript. MC and LM recruited and tested participants. LM contributed materials and analysis tools. MC, LM, and TK have revised and approved the manuscript. OW and TK identified patients for the study and acquired clinical data. JF and OW conceptualized and designed the study, interpreted the analysis, and have edited, revised, and approved the manuscript.

## Conflict of Interest Statement

NL, MC, and LM declare that they have no conflicts of interest. TK has served on scientific advisory boards for Roche, Genzyme, Novartis, Merck, and Biogen; has received conference travel support and/or speaker honoraria from WebMD Global, Novartis, Biogen, Sanofi, Genzyme, Teva, BioCSL, and Merck; and has received research support from Biogen. A/Prof. OW reports grants from Bayer, Australia, during the conduct of the study; personal fees from Bayer, Australia; grants from Biogen; grants and personal fees from Novartis; and personal fees from Genzyme, outside the submitted work. A/Prof. JF reports grants from Bayer, Australia, during the conduct of the study; grants from Biogen and Novartis, outside the submitted work. The reviewer LA declared a shared affiliation, though no other collaboration, with several of the authors NL, MC, TK, and JF to the handling Editor, who ensured that the process nevertheless met the standards of a fair and objective review.

## References

[1] Lublin FD Reingold SC Cohen JA Cutter GR Sørensen PS Thompson AJ Defining the clinical course of multiple sclerosis: the 2013 revisions. Neurology (2014) 83(3):278–86. 10.1212/WNL.0000000000000560 24871874 PMC4117366

[2] Polman CH Reingold SC Banwell B Clanet M Cohen JA Filippi M Diagnostic criteria for multiple sclerosis: 2010 revisions to the McDonald criteria. Ann Neurol (2011) 69(2):292–302. 10.1002/ana.22366 21387374 PMC3084507

[3] Yu CS Lin FC Liu Y Duan Y Lei H Li KC. Histogram analysis of diffusion measures in clinically isolated syndromes and relapsing-remitting multiple sclerosis. Eur J Radiol (2008) 68(2):328–34. 10.1016/j.ejrad.2007.08.036 17928182

[4] Fisher E Lee JC Nakamura K Rudick RA. Gray matter atrophy in multiple sclerosis: a longitudinal study. Ann Neurol (2008) 64(3):255–65. 10.1002/ana.21436 18661561

[5] Miller D Barkhof F Montalban X Thompson A Filippi M. Clinically isolated syndromes suggestive of multiple sclerosis, part 2: non-conventional MRI, recovery processes, and management. Lancet Neurol (2005) 4(6):341–8. 10.1016/S1474-4422(05)70071-5 15907738

[6] Roosendaal SD Schoonheim MM Hulst HE Sanz-Arigita EJ Smith SM Geurts JJG Resting state networks change in clinically isolated syndrome. Brain (2010) 133(6):1612–21. 10.1093/brain/awq058 20356855

[7] Henry RG Shieh M Okuda DT Evangelista A Gorno-Tempini ML Pelletier D. Regional grey matter atrophy in clinically isolated syndromes at presentation. J Neurol Neurosurg Psychiatry (2008) 79(11):1236–44. 10.1136/jnnp.2007.134825 18469033 PMC4827711

[8] Gallo A Rovaris M Riva R Ghezzi A Benedetti B Martinelli V Diffusion-tensor magnetic resonance imaging detects normal-appearing white matter damage unrelated to short-term disease activity in patients at the earliest clinical stage of multiple sclerosis. Arch Neurol (2005) 62(5):803–8. 10.1001/archneur.62.5.803 15883269

[9] Caramia F Pantano P Di Legge S Piattella MC Lenzi D Paolillo A A longitudinal study of MR diffusion changes in normal appearing white matter of patients with early multiple sclerosis. Magn Reson Imaging (2002) 20(5):383–8. 10.1016/S0730-725X(02)00519-2 12206862

[10] Sahraian MA Eshaghi A. Role of MRI in diagnosis and treatment of multiple sclerosis. Clin Neurol Neurosurg (2010) 112(7):609–15. 10.1016/j.clineuro.2010.03.022 20417027

[11] Fielding J Clough M Beh S Millist L Sears D Frohman AN Ocular motor signatures of cognitive dysfunction in multiple sclerosis. Nat Rev Neurol (2015) 11(11):637–45. 10.1038/nrneurol.2015.174 26369516

[12] Clough M Millist L Lizak N Beh S Frohman TC Frohman EM Ocular motor measures of cognitive dysfunction in multiple sclerosis I: inhibitory control. J Neurol (2015) 262(5):1130–7. 10.1007/s00415-015-7645-3 25851743

[13] Clough M Mitchell L Millist L Lizak N Beh S Frohman TC Ocular motor measures of cognitive dysfunction in multiple sclerosis II: working memory. J Neurol (2015) 262(5):1138–47. 10.1007/s00415-015-7644-4 25851742

[14] Fielding J Kilpatrick T Millist L Clough M White O. Longitudinal assessment of antisaccades in patients with multiple sclerosis. PLoS One (2012) 7(2):e30475. 10.1371/journal.pone.0030475 22319570 PMC3271102

[15] Fielding J Kilpatrick T Millist L White O. Antisaccade performance in patients with multiple sclerosis. Cortex (2009) 45(7):900–3. 10.1016/j.cortex.2009.02.016 19327763

[16] Fielding J Kilpatrick T Millist L White O. Multiple sclerosis: cognition and saccadic eye movements. J Neurol Sci (2009) 277(1–2):32–6. 10.1016/j.jns.2008.10.001 18977003

[17] Fielding J Kilpatrick T Millist L White O. Control of visually guided saccades in multiple sclerosis: disruption to higher-order processes. Neuropsychologia (2009) 47(7):1647–53. 10.1016/j.neuropsychologia.2009.01.040 19397859

[18] Pierrot-Deseilligny C Milea D Müri RM. Eye movement control by the cerebral cortex. Curr Opin Neurol (2004) 17(1):17–25. 10.1097/00019052-200402000-00005 15090873

[19] Leigh RJ Zee DS The Neurology of Eye Movements. 5th ed New York, NY: Oxford University Press (2015).

[20] Krauzlis RJ. Recasting the smooth pursuit eye movement system. J Neurophysiol (2004) 91(2):591–603. 10.1152/jn.00801.2003 14762145

[21] Jozefowicz-Korczynska M Pajor AM. Evaluation of the smooth pursuit tests in multiple sclerosis patients. J Neurol (2011) 258(10):1795–800. 10.1007/s00415-011-6014-0 21445600

[22] Frohman EM Frohman TC Zee DS McColl R Galetta S. The neuro-ophthalmology of multiple sclerosis. Lancet Neurol (2005) 4(2):111–21. 10.1016/S1474-4422(05)00992-0 15664543

[23] Rashbass C The relationship between saccadic and smooth tracking eye movements. J Physiol (1961) 159:326–38. 10.1113/jphysiol.1961.sp006811 14490422 PMC1359508

[24] Carl JR Gellman RS. Human smooth pursuit: stimulus-dependent responses. J Neurophysiol (1987) 57(5):1446–63. 3585475 10.1152/jn.1987.57.5.1446

[25] Schalen L. Quantification of tracking eye movements in normal subjects. Acta Otolaryngol (1980) 90(5–6):404–13. 10.3109/00016488009131742 7211334

[26] Orban de Xivry J-J Lefèvre P. Saccades and pursuit: two outcomes of a single sensorimotor process. J Physiol (2007) 584(1):11–23. 10.1113/jphysiol.2007.139881 17690138 PMC2277072

[27] Kolbe SC Kilpatrick TJ Mitchell PJ White O Egan GF Fielding J. Inhibitory saccadic dysfunction is associated with cerebellar injury in multiple sclerosis. Hum Brain Mapp (2014) 35(5):2310–9. 10.1002/hbm.22329 24038970 PMC6869843

[28] Felleman DJ Van Essen DC. Distributed hierarchical processing in the primate cerebral cortex. Cereb Cortex (1991) 1(1):1–47. 10.1093/cercor/1.1.1 1822724

